# Time Course of Upper Limb Function in Children with Unilateral Cerebral Palsy: A Five-Year Follow-Up Study

**DOI:** 10.1155/2018/2831342

**Published:** 2018-11-14

**Authors:** Katrijn Klingels, Sarah Meyer, Lisa Mailleux, Cristina Simon-Martinez, Jasmine Hoskens, Elegast Monbaliu, Geert Verheyden, Geert Verbeke, Guy Molenaers, Els Ortibus, Hilde Feys

**Affiliations:** ^1^KU Leuven, University of Leuven, Department of Rehabilitation Sciences, Leuven, Belgium; ^2^UHasselt, BIOMED, Rehabilitation Research Center (REVAL), Hasselt, Belgium; ^3^KU Leuven, University of Leuven, Leuven Biostatistics and Statistical Bioinformatics Centre, Leuven, Belgium; ^4^KU Leuven, University of Leuven, Department of Musculoskeletal Sciences, Leuven, Belgium; ^5^KU Leuven, University of Leuven, Department of Development and Regeneration, Leuven, Belgium

## Abstract

Knowledge on long-term evolution of upper limb function in children with unilateral cerebral palsy (CP) is scarce. The objective was to report the five-year evolution in upper limb function and identify factors influencing time trends. Eighty-one children (mean age 9 y and 11 mo, SD 3 y and 3 mo) were assessed at baseline with follow-up after 6 months, 1, and 5 years. Passive range of motion (PROM), tone, muscle, and grip strength were assessed. Activity measurements included Melbourne Assessment, Jebsen-Taylor test, Assisting Hand Assessment (AHA), and ABILHAND-Kids. At 5-year follow-up, PROM (*p* < 0.001) and AHA scores (*p* < 0.001) decreased, whereas an improvement was seen for grip strength (*p* < 0.001), Melbourne Assessment (*p* = 0.003), Jebsen-Taylor test (*p* < 0.001), and ABILHAND-Kids (*p* < 0.001). Age influenced the evolution of AHA scores (*p* = 0.003), with younger children being stable over time, but from 9 years onward, children experienced a decrease in bimanual performance. Manual Ability Classification System (MACS) levels also affected the evolution of AHA scores (*p* = 0.02), with stable scores in MACS I and deterioration in MACS II and III. In conclusion, over 5 years, children with unilateral CP develop more limitations in PROM, and although capacity measures improve, the spontaneous use of the impaired limb in bimanual tasks becomes less effective after the age of 9 years.

## 1. Introduction

Becoming independent in activities of daily living requires—amongst others—a smooth coordination between both hands. In children with unilateral cerebral palsy (CP), the occurrence of an early brain lesion elicits sensorimotor impairments in the contralateral upper limb. Such impairments compromise the development of upper limb function, which in turn restrains bimanual coordination [1]. Insights into the long-term evolution of upper limb function in these children are indispensable to inform parents about these restraints and to steer goal setting and treatment selection. Additionally, it may aid in distinguishing whether changes in upper limb function following an intervention program are attributable to therapy response or to natural change over time.

Thus far, four studies focused on long-term development of upper limb function in children with unilateral CP [2–5]. Holmefur et al. and Nordstrand et al. demonstrated improvements in the spontaneous use of the impaired hand during bimanual tasks in children aged between 18 months and 8 years or 12 years, respectively, who were followed over a period of 4.5 or 6 years, respectively [2, 3]. In contrast, two other studies did not find changes in bimanual performance nor grip efficiency in children with unilateral CP, assessed between 2 to 4 years up to 11 to 17 years of age [4, 5]. Clearly, contradicting results exist regarding the long-term developmental trajectory of bimanual performance while knowledge on the long-term evolution of motor impairments and unimanual capacity is scarce.

Moreover, the identification of characteristics to predict the longitudinal development of upper limb function in children with unilateral CP is crucial for improving prognoses and treatment planning. However, only limited information is available regarding which characteristics determine the long-term outcome of upper limb function in these children. Only one study previously reported the influence of age on spontaneous hand use demonstrating a rapid development at a young age, reaching a plateau between 2.5 and 8 years [3]. The age at which this plateau is reached depends on the initial manual ability of the child. Children with higher manual abilities develop at a faster rate, reaching their limits at a younger age, compared to children with lower manual abilities [2, 3]. Another factor that may influence the long-term evolution of upper limb function in children with CP is timing of the underlying brain lesion, broadly classified as congenital or acquired lesions. Acquired lesions are generally associated with more severe upper limb impairments compared to congenital brain lesions [6]. Moreover, in a one-year follow-up study of upper limb function, Klingels et al. showed that movement speed improved in children with congenital lesions, whereas children with acquired lesions remained stable [7].

In conclusion, there is a need for a better understanding of the long-term evolution capturing the different qualifiers of UL function as well as the identification of which child's characteristics adequately predict the long-term development of upper limb function assessed on body function and activity level according to the International Classification of Functioning, Disability and Health (ICF). Hence, the objectives of this study were (1) to report the evolution of upper limb function over five years in a large cohort of children with unilateral CP, including both measures at the level of body function and activities, and (2) to identify child's characteristics that influence these long-term time trends.

## 2. Materials and Methods

### 2.1. Participants

Children were recruited from the University Hospitals Leuven, special education schools, and one rehabilitation centre in Belgium between June 2007 and January 2008. Inclusion criteria were (1) a diagnosis of congenital or acquired unilateral CP and (2) age between 5 and 15 years. Acquired lesions were defined as lesions occurring in the developing infant brain between 28 days postnatally and three years [8]. Children were excluded if they had (1) insufficient cooperation to perform the assessments, (2) upper limb surgery, and (3) botulinum toxin-A injections in the upper limb within six months prior to baseline. In case a child received botulinum toxin-A injections in the upper limb during the study course, this child was excluded from the analysis of a specific time point if the injection was performed within six months prior to assessment. All children had access to the regular rehabilitation services. Ethical approval was obtained from the Ethics Committee of the University Hospitals Leuven (approval number: S50439), and parents signed a written informed consent form prior to participation.

### 2.2. Procedure

Children were assessed at baseline, at 6 months, and at 1 and 5 years of follow-up by two trained physiotherapists (KK, JH) routinely involved in the clinical evaluation of children with unilateral CP. All assessments were conducted at the place of recruitment. The results of the first year follow-up have been published in a previous paper [7].

### 2.3. Assessments

At baseline, age, gender, etiology (congenital or acquired lesion), and the Manual Ability Classification System (MACS) [9] were recorded. At each time point, the physiotherapists treating the children were asked to fill in a questionnaire on the intensity and content of the routine therapy the children received.

At body function level, a standardized test protocol was performed including upper limb passive range of motion (PROM), muscle tone, muscle strength, and grip strength. PROM of shoulder flexion, abduction, external and internal rotation, elbow extension, forearm supination, and wrist extension was measured using a goniometer. PROM values were dichotomized (0: no movement limitation, 1: movement limited by 10° or more compared to standard values). A sum score of these seven dichotomized scores resulted in a PROM total score between 0 and 7, with higher scores indicating more movement limitations. Muscle tone was evaluated in 11 muscle groups using the Modified Ashworth Scale (MAS), ranging from 0 to 4 [10]. A total score was calculated (0–44) including the muscle groups of the shoulder (adductors/abductors, extensors, and internal/external rotators), elbow (flexors/extensors), wrist (pronators and extensors/flexors), and fingers (flexors). To assess muscle strength, manual muscle testing (MMT) was administered in nine muscle groups with a score ranging from 0 to 5 [11]. A total sum score was calculated (0–45) for the muscle groups of the shoulder (flexors and abductors/adductors), elbow (extensors/flexors), forearm (supinators/pronators), and wrist (extensors/flexors). Grip strength was assessed with a Jamar® Inc. AUS dynamometer. The average of three consecutive maximum contractions was recorded for both hands. Also, the ratio of grip strength of the affected to the unaffected hand was calculated, expressed as a percentage, to eliminate the correlation with age [12]. Interrater and test-retest reliability of this protocol has been established [13].

At activity level, the *capacity* of the affected hand was assessed with the Melbourne Assessment of Unilateral Upper Limb function (Melbourne Assessment) and the Jebsen-Taylor hand function test. The Melbourne Assessment evaluates quality of movement in 16 functional unimanual tasks [14]. The total raw score (0–122) was converted to a percentage score, with higher scores indicating better capacity. The reported smallest detectable difference (SDD) for the Melbourne Assessment is 7.4% [15]. The Jebsen-Taylor hand function test measures manual dexterity in six unimanual tasks, by means of movement time expressed in seconds, with lower scores indicating better capacity [16]. Finally, *bimanual performance* was evaluated with the Assisting Hand Assessment (AHA) and ABILHAND-Kids questionnaire. The AHA, a Rasch-based performance scale, measures how effectively the affected hand is spontaneously used during performance of bimanual tasks [17]. Different test items, describing various object-related hand actions are scored on a 4-point scale rating the quality of performance. The raw scores from AHA version 4.4 (baseline, 6 months, and 1-year follow-up) and 5.0 (5-year follow-up) were converted through the Rasch analysis to logit scores varying between 0 and 100, with higher scores indicating higher ability levels. The SDD for the AHA is 5 AHA logits [18]. ABILHAND-Kids questionnaire is a Rasch-based inventory of 21 mostly bimanual activities that the parents were asked to judge as 0 (impossible), 1 (difficult), and 2 (easy) [19]. The raw scores were converted to logit scores. The reported SDD for the ABILHAND-Kids is 1.82 logits [20]. For all activity level assessments, high levels of reliability and validity have been established [19–23]. Videotapes of the Melbourne Assessment and AHA were scored by four experienced physiotherapists, all certified for AHA scoring. Prior to scoring, interrater reliability was verified in 10 children. Intraclass correlation coefficients between raters were 0.91 and 0.93 for the Melbourne Assessment and the AHA, respectively.

### 2.4. Statistical Analysis

Children's clinical and demographic characteristics were displayed as frequencies with percentages, means with standard deviations (SD), and medians with interquartile ranges (IQR), whichever appropriate. Linear mixed models (LMMs) were used to study longitudinal trends. Such models correct for the correlation amongst repeated observations within subjects using random effects. Also, when some observations are missing, LMMs still provide valid inferences, provided that missingness does not depend on unobserved outcomes (i.e., assuming missingness at random) [24]. To meet the distributional assumptions, an exponential transformation was used for the Melbourne Assessment and a natural logarithmic transformation for the Jebsen-Taylor test. Significant categorical time trends were further investigated with pairwise post hoc tests between baseline and 1-year follow-up and between 1- and 5-year follow-up. To identify factors that influence time trends, interaction terms between the factor time and the following factors were included in the models: age, gender, etiology, MACS, and botulinum toxin injections or participation in a modified CIMT intervention during the study course. To study the influence of age, three age groups were created: 5 to 7, 8 to 11, and 12 to 15 years old. To correct for multiple testing, pairwise post hoc time effects were tested at the 1% level of significance. All statistical analyses were performed using SAS version 9.4 (SAS Institute Inc., Cary, NC).

## 3. Results

### 3.1. Participants

Eighty-one children (43 boys and 38 girls) with congenital (*N* = 69, 85%) or acquired (*N* = 12, 15%) brain lesions were included. Mean age at first assessment was 9 years 11 months (SD 3 y and 3 m). Unilateral CP was left sided in 36 (44%) and right sided in 45 (56%) children. Forty-four (54%) children attended mainstream schools and 37 (46%) special education schools. According to the MACS, 29 (36%) children were classified as level I, 36 (44%) as level II, and 16 (20%) as level III. All children received regular physical therapy throughout the duration of the study, varying from one to five sessions weekly, with a median duration of 90 minutes per week (range 30–240 minutes). Of this time, therapists spent a mean time of 35% per session on upper limb treatment. Of the time spent on upper limb treatment, a mean of 41% of the time was dedicated to functional activities, 32% to stretching, 20% to strength training, and 7% to other aspects such as sensory training or electrical stimulation. The time spent on functional activities was almost equally divided between unimanual (48%) and bimanual activities (52%). Only three children ceased physiotherapy when reaching adulthood. Twenty-one children also received occupational therapy during the study course with a median duration of 45 minutes per week (range 20–90 minutes).

**Figure 1:**
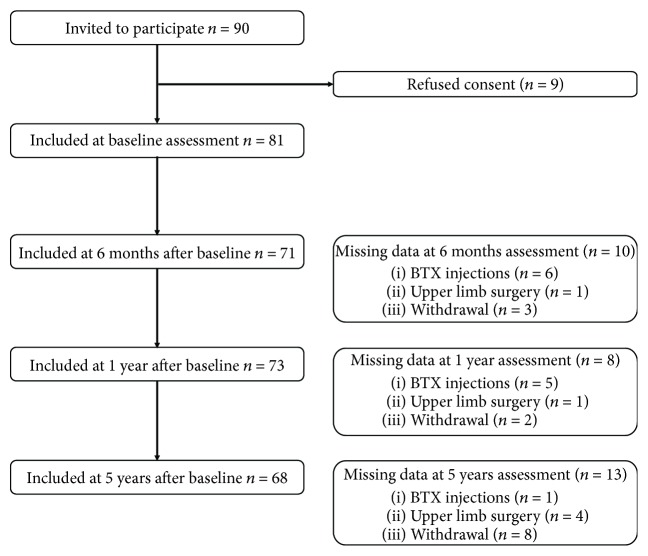
Number of children and details of missing data at all measurement points.

Figure 1 displays a flow chart detailing the number of participating children at the four assessments. During the study course, 10 children received botulinum toxin-A injections, of whom two received it twice. These children were excluded from the analysis of the next assessment if the injection was less than six months prior to the assessment. Between 1- and 5-year follow-up, 15 children participated in an intensive therapy study, including a home program of modified CIMT [25]. After this intensive training period, the children continued their regular physiotherapy sessions.

### 3.2. Time Course of Upper Limb Function over Five Years

**Figure 2:**
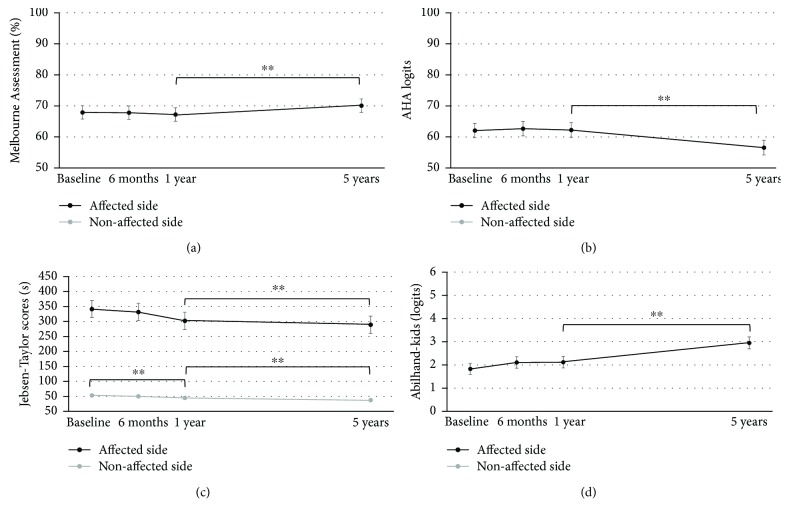
Mean and standard error estimates at baseline, 6 and 12 months, and 5 years for (a) Melbourne Assessment, (b) Assisting Hand Assessment (AHA), (c) Jebsen-Taylor test, and (d). ABIILHAND-Kids.

Table 1 shows the results of the LMM analysis. A significant deterioration over five years was noted for PROM (*p* = 0.008) and AHA scores (*p* < 0.001), whereas a significant improvement was seen for grip strength in both hands (*p* < 0.001), Melbourne Assessment (*p* = 0.002), Jebsen-Taylor test in both hands (*p* < 0.001), and ABILHAND-Kids (*p* < 0.001). Post hoc tests showed improvements between baseline and one-year follow-up for grip strength of the nonaffected hand (*p* < 0.001) and for the Jebsen-Taylor test in both hands (*p* < 0.001). Further, between one and five years, improvements were observed in grip strength at both sides (*p* < 0.001), Melbourne Assessment (*p* < 0.001), Jebsen-Taylor test (affected hand *p* < 0.001, nonaffected hand *p* = 0.002), and ABILHAND-Kids (*p* < 0.001). PROM and AHA scores, on the contrary, showed a significant deterioration between 1- and 5-year follow-up (PROM *p* = 0.028 and AHA *p* < 0.001). No significant time effects were found after five years for muscle tone (*p* = 0.17), muscle strength (*p* = 0.86), and the ratio between grip strength of the affected versus nonaffected hand (*p* = 0.92). Figures 2(a)–2(d) show the time trends of the activity outcome measures.

For the outcome measures with reported SDDs, we explored whether individual change scores between baseline and 5-year follow-up exceeded the SDD threshold (7.4%). For the Melbourne Assessment, 13 (19%) children improved more than 7.4%, 51 children (75%) remained stable, and four children (6%) deteriorated more than 7.4%. In contrast, on the AHA, 13 (20%) children improved more than 5 AHA logits, 17 (27%) remained stable, and 34 (50%) deteriorated with at least 5 AHA logits. Finally, for the ABILHAND-Kids, 15 (31%) children improved above the SDD threshold of 1.8 logits, 33 (67%) children remained stable, and only one (2%) child deteriorated over five years.

### 3.3. Influencing Factors

**Figure 3:**
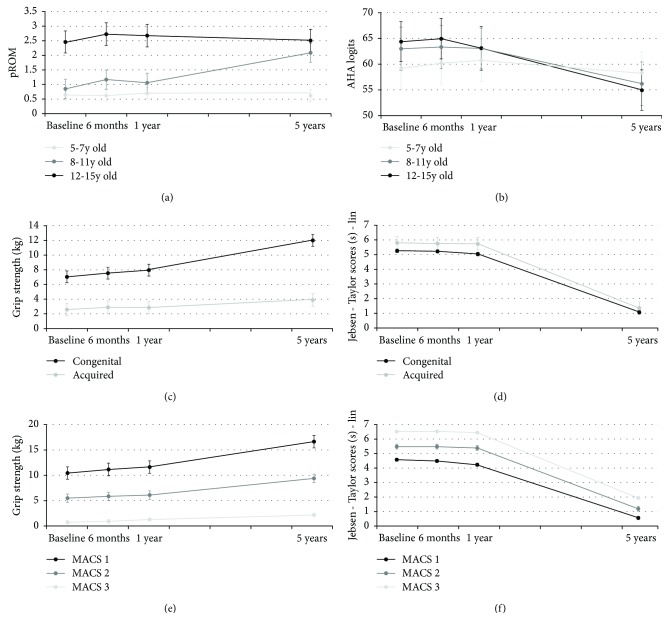
Means and standard error estimates at baseline, 6 and 12 months, and 5 years for the three age groups for (a) passive range of motion (PROM) and (b) Assisting Hand Assessment (AHA); for the two etiology groups for (c) grip strength on the affected side (AS) and (d) Jebsen-Taylor scores on AS; and for the three Manual Ability Classification System levels (MACS) for (e) grip strength on AS and (d) Jebsen-Taylor scores on AS.

Age had a significant influence on the time evolution of the PROM (*p* < 0.001), with children between 8 and 11 years old at baseline acquiring more movement limitations between 1- and 5-year follow-up (Figure 3(a)). Age also significantly influenced the evolution of AHA scores (*p* = 0.003), with younger children being stable over time but older children from the age of 9 years, showing a decrease in AHA scores (Figure 3(b)). Secondly, gender influenced the evolution of grip strength, which improved significantly more in boys (*p* < 0.001). Etiology also influenced evolution of grip strength and Jebsen-Taylor scores (both *p* < 0.001), which improved significantly more in children with congenital lesions compared to acquired lesions (Figures 3(c) and 3(d)). Furthermore, MACS levels influenced the evolution of grip strength (Figure 3(e)) and Jebsen-Taylor scores (Figure 3(f)), with better improvements in grip strength (*p* < 0.001) and Jebsen-Taylor scores (*p* < 0.001) in children with MACS level I.

**Figure 4:**
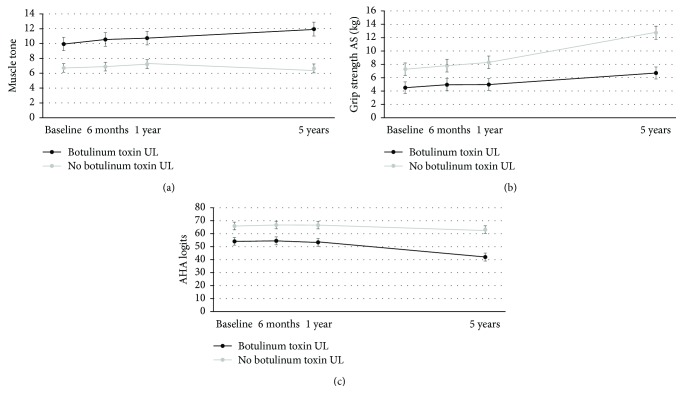
Means and standard error estimates at baseline, 6 and 12 months, and 5 years for the groups of children who did or did not receive botulinum toxin injections during the study course for (a) muscle tone, (b) grip strength on the affected side (AS), and (c) Assisting Hand Assessment (AHA).

Children who received botulinum toxin injections during the study course showed significantly more increase in muscle tone (*p* = 0.01), less increase in grip strength at the affected side (*p* = 0.0006), and more pronounced decline in AHA scores compared to children who did not receive injections (*p* < 0.0001) (Figures 4(a)–4(c)). Finally, the participation in a modified CIMT program did not influence the evolution of any of the activity measures (*p* > 0.08).

## 4. Discussion

This study aimed to map the 5-year time course of upper limb function and the influencing factors in children with unilateral CP according to the ICF body function and activity level. Results showed increased limitations in PROM mainly from the age of 9 years onwards. Furthermore, grip strength and unimanual capacity improved over time, mostly in mildly affected children. On the contrary, the spontaneous use of the affected upper limb in bimanual activities became less effective, again from the age of 9 years onwards.

Results at body function level showed more PROM limitations over time, mainly developing in children aged 9 years and older, while this process stabilizes around 14-15 years of age. Visual inspection showed most pronounced limitations for wrist extension. This confirmed the results of a recent study that reported a twofold increase in skeletal muscle stiffness of the wrist and finger flexors in children with unilateral and bilateral CP compared to typically developing children [26]. The cause of the increased stiffness is however yet unknown, though it can be hypothesized that it is attributed to an increased content of intramuscular collagen [27], together with an increased amount of connective tissue around fiber bundles, i.e., a thickening of the perimysial extracellular matrix [28]. These results imply that current methods to lengthen wrist and finger flexor muscles are of utmost importance to be applied in this age group. This may include stretching, use of splints, and botulinum toxin injections followed by intensive therapy and surgical interventions, e.g., tendon transfer surgery.

Additionally, grip strength increased over time both in the affected and nonaffected hand. Improvements were mainly seen in children with MACS level I and congenital lesions. It seems that children with better hand function are more likely to improve over time [2, 3, 7, 29]. The grip strength ratio between the affected and nonaffected hand remained stable around 40%, implying that grip strength increased at the same rate in both hands.

At activity level, significant improvements were found in unimanual capacity, based on the Melbourne Assessment and Jebsen-Taylor scores. Again, most improvements were seen in children with MACS level I and with congenital lesions. For typically developing children with comparable ages, Taylor et al. reported an age-related 10% reduction in time to perform the Jebsen-Taylor test (i.e., from 31.5 seconds at 10-11 years to 28.4 seconds at 15–19 years) [16]. The mean time to perform the test in our sample of children with unilateral CP decreased with 15% over five years, which may likely be of clinical significance.

Surprisingly, despite improvements in unimanual capacity, deterioration was seen in bimanual performance. From the age of 9 years onwards, children seem to use the affected arm less and less efficiently in bimanual activities, which is also a common complaint of parents. This finding is in accordance with the study of Fedrizzi et al. reporting less improvement in spontaneous hand use than in grip assessment between the age of 4 and 11 years [4]. We hypothesize that several factors may contribute to this deterioration in bimanual performance such as the presence of sensory deficits [1], mirror movements [30], and developmental disregard [31]. In the study of Nordstrand et al. children with unilateral CP showed a rapid development of bimanual performance at a young age and reached 90% of their estimated limit between 30 months and 8 years [3]. These authors attempted to investigate whether there was a decline in hand function, as the children approached 12 years of age. However, results were inconclusive because of too few data in this age group [3]. The novel finding of decline in bimanual function in our study has important clinical implications. To improve bimanual performance, a wide range of evidence-based therapy models can be applied such as CIMT, bimanual therapy, or combined models [32]. These models involve intensive blocks of goal-directed, skills-based practice. High-level evidence has shown that CIMT is effective for improving unimanual capacity brought about by implicit learning [33]. However, CIMT is not the most optimal modality to target explicit learning required for learning how to use both hands together in daily skills. Therefore, from the age of 9 years onwards, it may be more effective to organize intensive training focusing on bimanual performance. According to the motor learning principle of training specificity implying that “you progress to what you actually practice,” learning bimanual skills may be best achieved through practice of bimanual tasks [33].

Despite the decrease in bimanual performance as tested with the AHA, a significant improvement was found in ABILHAND-Kids scores over five years. We assume that these differences may be related to the nature of the tests. The AHA is a structured play session of bimanual activities in which the use of the assisting hand is scored, for example, how well the child moves his upper arm or forearm, whether he varies his type of grasp, or how he regulates his grip force. The ABILHAND-Kids on the other hand rates the perception of the parent on the ease or difficulty of the child in performing daily life activities. This does not take into account how the task is performed, whether this is one handed or with the help of other body parts such as their teeth to open a bag of chips or their arm to fixate a bottle to unscrew it. We assume that with maturation, children improve their motor learning and planning and adopt compensation strategies to perform ADL activities with more ease. This results in higher independency in daily life activities.

Children in our study had access to local services. This access includes regular check-ups and a wide range of physiotherapy and occupational therapy interventions. A subsample of 15 children also followed a home-based modified CIMT program between the period of one and five-year follow-up. Significant improvements in bimanual performance were reported immediately after CIMT and were retained at 10-week follow-up [25]. However, follow-up results showed that around three years later, the time course of bimanual performance did not differ between the group that did or did not receive the CIMT program. This may imply that repetitive boosts of therapy are needed to attain long-term improvements.

This study excluded children who received botulinum toxin injections within 6 months prior to the time point testing to rule out immediate effects of the injections. After six months, these children were enrolled again in the study, although we acknowledge that long-term effects of botulinum toxin injections might exist [32]. We did not exclude these children from further follow-up as (1) botulinum toxin can be considered as common care in our settings and (2) excluding these children would have induced selection bias and would result in a nonrepresentative sample of children with unilateral CP. Indeed, further data inspection showed that the children who received botulinum toxin injections during the study course were mostly classified as MACS levels II and III and showed pronounced deficits in muscle tone, grip strength, and bimanual performance at baseline. This may explain why these children also showed more deterioration in function compared to children who did not receive injections. This confirms our statistical assumption for linear mixed models that the missingness of these data points does not depend on unobserved outcomes but on observed outcomes, namely, MACS levels and assessments of muscle tone, grip strength, and bimanual performance.

This study included a large cohort of children with unilateral CP and a standardized set of reliable outcome measures at body function and activity level. Results were based on robust statistical modelling taking inevitable drop-outs into account. However, some limitations need to be recognized. First, for body function measures of spasticity and strength, ordinal rating scales were used, which are dependent on subjective interpretation. Therefore, great efforts were pursued to maximize standardization. Ordinal scales might also be less sensitive to subtle changes in muscle tone. As an alternative, future study should include quantitative measures such as dynamometers or instrumented spasticity measures [34] that might be more sensitive to change and will improve our understanding of upper limb function evolution in this population. Secondly, this study was based on a convenience sample, recruited in different centres. During the study course, all children received routine therapy and a subset received CIMT or botulinum toxin injections. In our health care system in Belgium, routine physiotherapy is commonly organized in distributed practice with one to five individual physiotherapy sessions per week. Our results, therefore, cannot be generalized to children receiving other service conditions, such as short boosts of intensive therapy. Finally, we acknowledge that also other neurological biomarkers, such as corticospinal tract reorganization, may influence longitudinal development of upper limb function, which warrants further investigation.

## 5. Conclusions

The novel findings from this large longitudinal study are that although different capacity measures improve over time, the spontaneous use of the affected upper limb in bimanual tasks decreases and becomes less effective from the age of 9 years onwards. Additionally, children with unilateral CP develop more limitations in PROM in the upper limb, more specifically for wrist extension, over a 5-year time period. These novel insights in the spontaneous evolution of upper limb function in children with unilateral CP and the factors that influence these time trends can provide guidance in delineating treatment priorities.

## Figures and Tables

**Table 1:** Results of the linear mixed models analysis: mean (SE) estimates of outcome measures at baseline, 6 and 12 months, and 5 years.

	Baseline	6 months	1 year	5 years	*p* value^a^
PROM (0–7)	1.30 (0.2)	1.47 (0.2)	1.46 (0.2)	1.76 (0.2)	**0.008**
Muscle tone (0–44)	7.75 (0.53)	8.06 (0.54)	8.37 (0.53)	8.3 (0.54)	0.17
Muscle strength (0–45)	31.91 (0.54)	32.00 (0.55)	31.85 (0.55)	31.73 (0.55)	0.85
Grip strength					
Absolute scores AS (kg)	6.39 (0.72)	6.87 (0.74)	7.23 (0.73)	10.87 (0.75)	**<0.0001**
NAS (kg)	15.88 (1.03)	17.31 (1.05)	18.12 (1.05)	25.86 (1.06)	**<0.0001**
Ratio (%)	40.0 (3)	39.0 (3)	40.0 (3)	40 (3)	0.92
Melbourne Assessment (%)	67.92 (2.15)	67.82 (2.16)	67.24 (2.16)	70.09 (2.17)	**0.002**
AHA (logits 0–100)	62.12 (2.33)	62.74 (2.35)	62.29 (2.34)	56.58 (2.36)	**<0.0001**
Jebsen-Taylor test (s)					
AS	341.29 (28.74)	331.53 (28.86)	302.1 (28.87)	289.09 (29.93)	**<0.0001**
NAS	53.3 (3.34)	50.39 (3.42)	44.96 (4.43)	37.52 (3.46)	**<0.0001**
ABILHAND-Kids (logits)	1.83 (0.24)	2.11 (0.25)	2.12 (0.25)	2.95 (0.26)	**<0.0001**

PROM: passive range of motion; AHA: Assisting Hand Assessment; AS: affected side; NAS: nonaffected side; SE: standard error; ^**a**^: linear mixed models

## Data Availability

The underlying data related to this submission is available by request to the first author.
